# Posterior reversible encephalopathy syndrome and takotsubo cardiomyopathy associated with lenvatinib therapy for thyroid cancer: a case report and review

**DOI:** 10.18632/oncotarget.25606

**Published:** 2018-06-15

**Authors:** Young Kwang Chae, Lauren Chiec, Scott K. Adney, Josh Waitzman, Ricardo Costa, Benedito Carneiro, Maria Matsangou, Mark Agulnik, Peter Kopp, Frank Giles

**Affiliations:** ^1^ Feinberg School of Medicine, Northwestern University, Chicago, IL 60611, USA; ^2^ Robert H. Lurie Comprehensive Cancer Center of Northwestern University, Chicago, IL 60611, USA; ^3^ H. Lee Moffitt Cancer Center and Research Institute, Tampa, FL 33612, USA; ^4^ Lifespan Cancer Institute, Providence, RI 02906, USA

**Keywords:** lenvatinib, posterior reversible encephalopathy syndrome, takotsubo cardiomyopathy, thyroid cancer

## Abstract

As immunotherapies including tyrosine kinase inhibitors become more widely used for the treatment of a variety of malignancies, it is important for prescribers and patients to understand the potential adverse effects associated with these drugs. It is especially important to understand the potentially fatal side effects associated with these drugs to further determine risk factors for their development. The review presents a case of posterior reversible encephalopathy syndrome with concomitant Takotsubo cardiomyopathy, associated with use of lenvatinib therapy for thyroid cancer. It discusses the interventions performed and outcome. Potential mechanisms for development of these rare adverse effects, as well as cases in which these adverse effects are seen with use of other tyrosine-kinase inhibitors will be presented. It is important to continue to report these side effects, and further studies are needed to elucidate potential risk factors for their development, as well as to determine prognosis after development.

## INTRODUCTION

Posterior reversible encephalopathy syndrome (PRES), is a neuroradiologic phenomenon that manifests with headache, confusion, focal deficits, and can progress to epilepsy, coma, and result in death. Since 1996 when the constellation of neurologic and radiologic findings was first described as the posterior leukoencephalopathy syndrome, it has been historically associated with hypertensive emergency and immunosuppression [1]. With the advent of newer targeted therapies for cancer, this syndrome has emerged as a class side effect of tyrosine kinase inhibitors (TKIs), albeit uncommon. This case report describes a case of PRES associated with lenvatinib use with concomitant Takotsubo cardiomyopathy (TC), as well as a review of TKI-associated PRES and TC.

### Posterior reversible encephalopathy syndrome

PRES can also be referred to as reversible posterior leukoencephalopathy syndrome (RPLS). The edema associated with PRES that is thought to account for the patient's altered mentation and other neurologic symptoms is a consequence of cerebral blood flow autoregulation. The balance of vasoconstriction and vasodilation in response to changing cerebral perfusion pressures works to keep the cerebral blood flow fairly constant. It is thought that when this autoregulatory mechanism breaks down, either in response to rapid changes in cerebral perfusion pressure or a systemic inflammatory response, intravascular fluid leaks out causing cerebral edema that ultimately leads to PRES [2, 3]. Three radiologic patterns of PRES have been described depending on the location and extent of white-matter involvement, usually resulting in hyper-intense T2 and FLAIR sequences in a brain MRI. The parieto-occipital pattern involves the posterior brain, and is the origin of the acronym for PRES. A hemispheric watershed pattern has also been described in the literature, with involvement of the border zones between the anterior cerebral artery and the middle cerebral artery, thus manifesting in edematous changes to the frontal, parietal and occipital lobes. A more localized pattern is seen with involvement of the bilateral superior frontal sulci. In atypical cases involvement of other regions has been described, including the cerebellum and brainstem, but these are usually associated with parieto-occipital changes [2].

Treatment is largely supportive and symptom targeted. For those cases presenting with elevated blood pressure, reduction of the mean arterial pressure by 25% in the first several hours is usually suggested. If a drug cause for the syndrome can be isolated, the offending drug should be stopped. Patients presenting with seizures may require antiepileptic drug loading, and patients with severely altered mental status may require intubation for airway protection. Complications can include localized ischemia of involved brain regions, as well as intracranial hemorrhage, and frequent neurological monitoring is paramount.

As suggested by the condition name including the word “reversible”, most case reports of PRES suggest that signs and symptoms resolve within days to weeks. However, in one case series of 22 patients, 6 cases were fatal and some survivors suffered permanent neurologic damage [4]. Given the relative rarity of this disorder, prognostic indicators have been limited. One study suggests that MRI findings consistent with cytotoxic compared to vasogenic edema may imply a worse prognosis, as does greater brain involvement.

### Takotsubo cardiomyopathy

Takotsubo cardiomyopathy, sometimes referred to as stress cardiomyopathy, is a generally reversible phenomenon of cardiac dysfunction clinically mimicking a myocardial infarction. It is associated with an increase in cardiac biomarkers such as troponins, and can manifest with electrocardiogram changes including lead-contiguous ST-segment elevations or depressions. Takotsubo cardiomyopathy is best diagnosed with an echocardiogram, which shows the characteristic apical ballooning and midventricular akinesis [5].

Treatment of TC is also largely supportive, with conservative therapies and guideline-directed treatment of congestive heart failure until recovery. There are currently no specific data or guidelines demonstrating optimal medical management of TC. As patients with severely reduced ejection fractions are at risk for the development of left ventricular (LV) thrombus, this should be treated promptly with anticoagulation if identified. It may also be beneficial to treat patients with an LV EF of less than 30% with anticoagulation until resolution of wall motion abnormalities to prevent LV thrombus, although there is no randomized data supporting this.

Most patients with TC recover, although there is significant risk of in-hospital complications, including cardiogenic shock and death. One study suggested that the risk of in-hospital complications was similar to that of patients with acute coronary syndrome [6]. However, patients who survive the acute episode generally recover systolic function within four weeks [7].

## CASE PRESENTATION

The patient is a 58-year-old non-smoking female with a history of recurrent papillary thyroid cancer first detected in the 1970s, who throughout her course underwent several surgical resections, repeated therapy with radioactive-iodine, and external beam radiation therapy (EBRT) (original history, cumulative radioiodine dose and EBRT dose unavailable). She presented again in 2015 with recurrent thyroid cancer as well as newly diagnosed breast cancer and liver metastases of unknown origin. She was hospitalized for unexplained fevers, failure to thrive, and symptomatic management of disease progression. Prior to this hospitalization, she had undergone an extensive infectious disease workup with an attempt to isolate the cause of her fevers up to 104 degrees Fahrenheit over the preceding three months. No clear etiology was identified. Empiric antibiotics were eventually discontinued, and the presumed diagnosis of tumor fever was made. On discharge, she followed up in the oncology clinic to start lenvatinib, a TKI approved for radioiodine-refractory thyroid cancer. Hours after her first dose of lenvatinib, she was taken to the hospital due to two witnessed generalized tonic-clonic seizures. At home she was noted to have shaking of her arms with repetitive movements of her head, lasting about three minutes and followed by post-ictal confusion, with no tongue biting or incontinence noted. She was taken to the emergency department where she had another witnessed seizure that resolved under therapy with lorazepam. The patient was then transferred from the emergency department to a different medical center. Although her presenting blood pressure was not available for review, her blood pressure was noted to be 158/99 mmHg immediately prior to her transfer.

**Figure 1:**
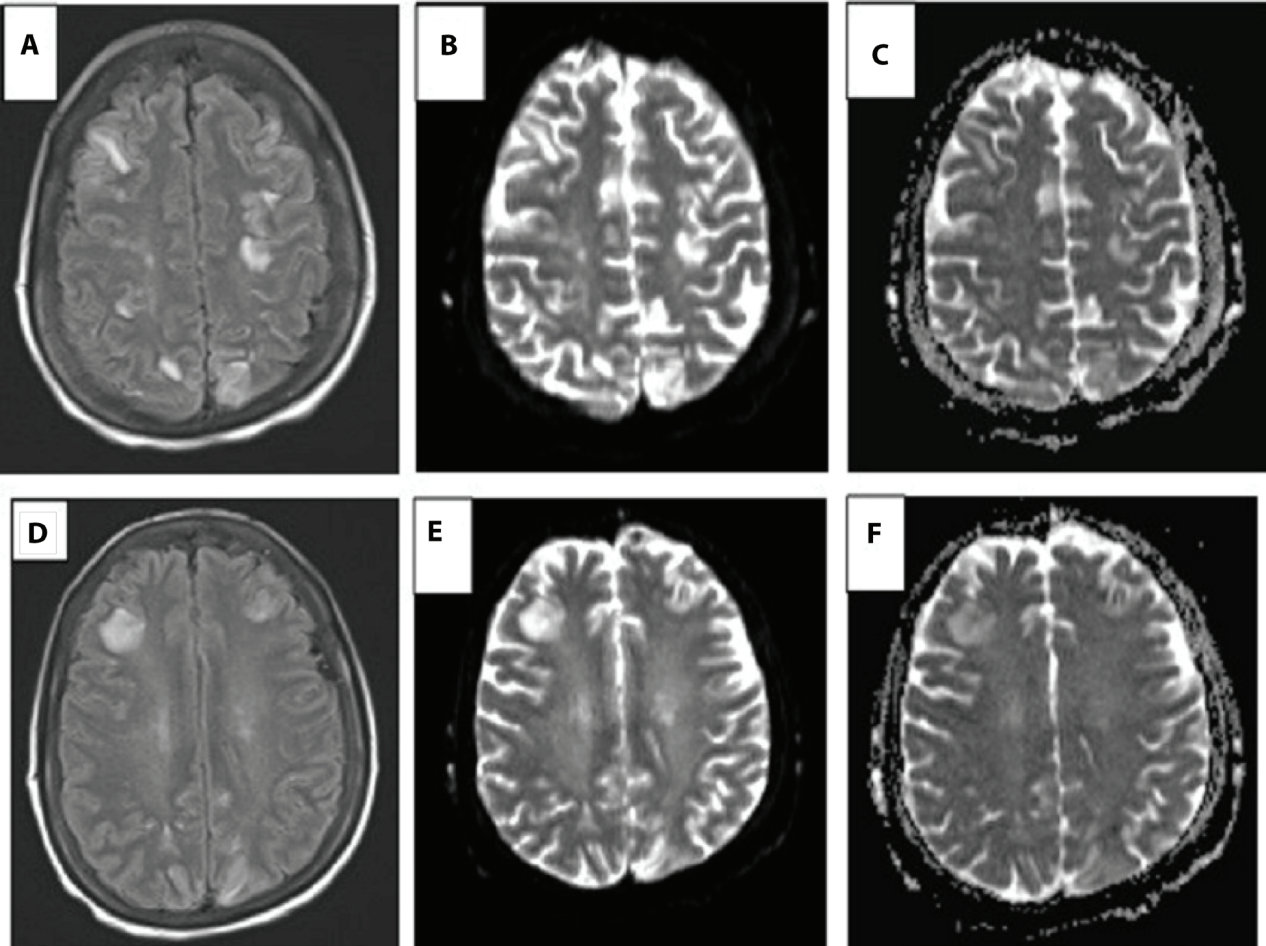
Brain MRI images consistent with PRES or posterior reversible leukoencephalopathy syndrome after lenvatinib treatment (**A**–**C**) demonstrate diffuse hyperintensities in the frontal and parieto-occipital cortices. (**D**–**F**) demonstrate localized edema around the right frontal lesion. (A, D) Fluid-attenuated inversion recovery imaging sequence (FLAIR). (B, E) Diffusion weighted imaging (DWI). (C, F) Apparent diffusion coefficient imaging (ADC)

The patient was admitted to the medical intensive care unit, and given persistent seizures, required intubation for airway protection. Physical exam was limited due to sedation. Brain MRI revealed multifocal white matter edema affecting the occipital and parietal lobes with patchy gadolinium enhancement, in a predominantly posterior distribution, but also with frontal lobe lesions with associated vasogenic edema and patchy enhancement (Figure 1). A small, acute infarct in the left centrum semiovale on diffusion weighted imaging was also noted. She developed a fever early in this hospitalization and extensive work up for infectious causes revealed right lower lobe pneumonia that was treated with vancomycin and cefepime. Given her fevers and altered mental status, as well as small acute stroke seen on MRI, patient underwent lumbar puncture on hospital day 1 to evaluate for signs of meningitis or systemic vasculitis. Her CSF studies were not consistent with those diagnoses, with 1–4 white blood cells per UL, 0–1 red blood cells per UL, glucose of 65 mg/dL (normal 40–70 mg/dL), protein of 46 mg/dL (normal 15–45 mg/dL) and gram stain and culture which was negative for growth. CSF was also negative for oligoclonal bands, herpes simplex virus, Ebstein-Barr virus, John Cunningham (JC) virus, cytomegalovirus, varicella zoster virus, and negative for growth of mycobacteria. Additionally, head and neck CT angiogram did not demonstrate any hemodynamically significant stenosis. Based on these clinical findings of confusion and progression to mental status depression, a presumed diagnosis of PRES was made. It was thought that the small stroke seen on MRI was a separate event and was not contributing to her current clinical picture. On hospital day 2, she required norepinephrine due to mixed distributive and cardiogenic shock. A transthoracic echocardiogram (TTE) showed an ejection fraction (EF) of 36%, decreased from 62% one month prior, with regional wall motion abnormalities, moderate to severe hypokinesis in all basal to mid-ventricular segments, and hyperdynamic apical segments. These findings were consistent with stress cardiomyopathy or reverse Takotsubo cardiomyopathy. She was weaned off vasopressors and extubated successfully on day 4. For her stress cardiomyopathy with tachycardia, she was started on captopril and metoprolol.

A repeat MRI one week later showed marked improvement in the size and number of areas of T2/FLAIR signal hyperintensity in the supratentorial cortex and near-complete resolution of the abnormal T2/FLAIR signal in the cerebellar hemispheres, further consistent with PRES. Repeat TTE at that time showed improvement of systolic function with an EF of 56% and no wall-motion abnormalities. She was continued on lacosamide and advised to follow up with neurology for epilepsy management. She was advised to not take lenvatinib. She remained hemodynamically stable on the oncology ward, however she had a non-resolving lactic acidosis without systemic signs of poor organ perfusion that was attributed to her cancer progression. The patient was discharged and ultimately died under hospice-care one week later.

## DISCUSSION

### Drug-induced PRES

Although the pathophysiology of PRES has not been entirely elucidated, the syndrome has been associated with acute hypertension, vascular or autoimmune disease, preeclampsia or eclampsia, renal disease, sepsis and various drugs, specifically with immunosuppressive medications. Drugs that target receptors regulating vascular permeability or altering immune response have been suspected to induce PRES [8]. In one combined prospective and retrospective study of 120 cases of PRES (113 patients), 19% of cases were associated with cytotoxic medications [9]. In addition, 42% of the study patients were being treated with immunosuppressive medications, including cyclophosphamide, tacrolimus, cyclosporine, mycophenolate, and less commonly bevacizumab, rituximab, vincrinstine, methotrexate, hydroxychloroquine, 5-fluorouracil, sirolimus, thalidomide, gemcitabine, paclitaxel, carboplatin, sorafenib, infliximab and hydroxyurea.

### TKI-induced PRES

TKI-associated PRES, while rare, is emerging as a serious adverse event related to treatment. Multiple case reports have described PRES associated with use of these agents. Interference with the vascular endothelial growth factor (VEGF) pathway is suspected to be a common theme in the pathophysiology. In a review of published case reports, TKIs with VEGF receptor antagonism such as sorafenib, sunitinib, axitinib, pazopanib, regorafenib, and cediranib have been associated with the development of PRES [2, 10–30]. Table 1 summarizes case reports of PRES attributed to TKI use, extending the findings of Myint *et al.* [22]. Interestingly, the TKI bortezomib, which is a proteasome inhibitor, has also been reported to be associated with PRES, and bevacizumab, a monoclonal antibody against VEGF receptor has also been reported [31–37]. The B-raf inhibitors, including vemurafenib, have also been found to induce PRES [26, 38]. At this time, it is unclear whether other TKIs have been associated with PRES without having been reported, or whether this association is not present in some of them. While rare, it appears that many different TKIs share the ability to induce PRES, and that VEGF antagonism may play a role, such that clinicians should be aware of this potential side effect when prescribing these novel medications.

**Table 1:** Tyrosine kinase inhibitors reported to be associated with the development of posterior reversible encephalopathy syndrome

TKI implicated in PRES	Number of patients reported	Targets	MRI enhancement pattern	Symptoms	References
Sorafenib	3	CRAF, VEGFR-1, VEGFR-2, VEGFR-3, PDGFR-β, Raf-1, BRAF, mutant BRAF, KIT, FLT-3, RET	Occipital, frontal	Headache, vision loss, seizures, loss of consciousness, dizziness, hallucinations, hypersalivation, gait disturbance, fever, weakness	[26, 27, 54]
Sunitinib	7	VEGFR-1, VEGFR-2, VEGFR-3, PDGFR-α, PDGFR-β KIT, FLT-3, CSF-1R, RET	Occipital, parietal, cerebellum, parieto-temporal, parieto-occipital	Dizziness, loss-of consciousness, confusion, seizures, headache, visual changes, vision loss, asthenia, unsteady gait, upper extremity weakness, dysdiadokinesia, dysmetria, verbal loss	[10, 13, 15–17, 24, 25, 55]
Pazopanib	6	VEGFR-1, VEGFR-2, VEGFR-3, PDGFR-α, PDGFR-β, FGFR-1, FGFR-3, KIT, Itk, Lck, c-Fms	Occipital, parietal, temporal, thalamic, frontal	Seizures, vision loss, headache, vomiting, unsteady gait, left arm paresis, dizziness, anasarca	[12, 23, 28–30, 56]
Regorafenib	1	RET, VEGFR-1, VEGFR-2, VEGFR-3, KIT, PDGFR-α, PDGFR-β, FGFR-1, FGFR-2 TIE2, DDR2, Trk2A, Eph2A, RAF-1, BRAF, BFAF^V600E^ SAPK2, PTK5, Ab1	cerebellar, posterior frontal, parietal, occipital	Seizures, agitation, altered mental status	[22, 57]
Axitinib	1	VEGFR-1, VEGFR-2, VEGFR-3	Occipital	Seizure, loss of consciousness	[18, 58]
Cediranib	3	VEGFR, KDR, Flt-1	cerebral peduncles, pons, medulla, thalami	Confusion, fluctuating consciousness	[11, 20, 21, 59]
Vemurafenib	1	BRAF^V600E^, CRAF, ARAF, wild-type BRAF, SRMS, ACK1, MAP4K5, FGR	Unable to perform MRI	Upper extremity swelling, confusion, vision loss, partial seizure	[38, 60]
Lenvatinib	6	VEGFR-1, VEGFR-2, VEGFR-3 FGFR-1, FGFR-2, FGFR-3, FGFR-4, PDGFR-α, KIT, RET	occipital, parietal, frontal	Seizures, confusion, shock	[41, 42], Authors’ case

a. Targets for the tyrosine kinase inhibitors were listed based on their documentation in the respective package insert.

b. Cediranib is not currently FDA-approved for use. Target information for cediranib was obtained from reference 59.

Lenvatinib is a newer targeted therapy tyrosine kinase inhibitor that has shown significant efficacy in radio-iodine refractory thyroid cancer [39]. The trial that led to FDA approval demonstrated an increase in progression-free survival in patients taking lenvatinib over placebo, however there was an increased risk of adverse events in the drug study arm. The original trial reported one case of PRES out of the entire cohort receiving lenvatinib (392 patients), and the current package insert states that 3 cases of PRES (called reversible posterior leukoencephalopathy syndrome here) were reported across clinical studies of 1108 patients who received lenvatinib [40, 41]. In addition, there has also been a published case report of a patient who developed PRES after treatment with lenvatinib for anaplastic thyroid cancer [42]. In that report, the patient developed hypertension 19 days after initiation of treatment, and developed true PRES 30 days after initiation, in contrast to the patient presented in the current report who developed symptoms after the initial dose of lenvatinib. This suggests that further reports are needed to better understand when patients may be at the greatest risk for development of PRES after initiation of lenvatinib.

### Drug-induced Takotsubo cardiomyopathy

The pathogenesis of stress cardiomyopathy is unclear, but animal studies suggest that catecholamine surges during periods of physical or emotional stress can exacerbate cardiac dysfunction, either by causing vasoconstriction of coronary vasculature or through a direct cytotoxic effect on the cardiac myocytes [5]. It is therefore not surprising that sympathomimetic drugs are commonly listed as causative agents of stress cardiomyopathy, including epinephrine, dobutamine, and ephedrine. Other drugs that have been associated with drug-induced Takotsubo cardiomyopathy include ergonovine, oxymetazoline, atropine, duloxetine, nortriptyline, venlafaxine, levothyroxine, and potassium chloride. The withdrawal of metoprolol and oxycodone have also been associated with the development of TC. TC has been associated with chemotherapeutic agents and hematologic agents, specifically 5-fluorouracil, combretastatin, pazopanib, and anagrelide, as well as a case report of a patient treated with cytarabine and daunorubicin who developed TC [5, 43]. At this time, the mechanism of TC due to nonsympathomimetic drugs is unclear. It has been suggested that TC may develop from the catecholamine surges and stress associated with the process of undergoing treatment for malignancy. Additionally, for TKIs such as pazopanib, it has been suggested that VEGF antagonism may play a mechanistic role through modulation of nitric oxide and catecholamine effects, with VEGF antagonism reducing nitric oxide levels and causing an increased response to catecholamines in some organs [5, 43–45].

### TKI-associated takotsubo cardiomyopathy

Cardiotoxicity and cardiomyopathy have been associated with TKI use. In one report of sunitinib and sorafenib, one patient each developed a reversible cardiomyopathy which was thought to be associated with the TKI use, however it is unclear from that report whether these patients had echocardiographic findings consistent with TC [46]. Similar findings were reported in another case of a patient treated with sunitinib, with reversible cardiomyopathy demonstrated, but no discussion of TC [47]. In another study of a cohort of 75 patients with gastrointestinal stromal tumors receiving sunitinib, 11% were found to have a cardiovascular event, most commonly New York Heart Association class III-IV congestive heart failure. Left ventricular dysfunction and symptoms improved in 5/6 of the patients who developed congestive heart failure. This study however, suggested that sunitinib was associated with direct cardiotoxic effects, but it did not mention if the patients had any characteristic findings of TC [48]. There has also been a report of toxic cardiomyopathy associated with vandetanib, again without demonstration of classic TC findings and without reversibility, as the patient passed away secondary to a fatal arrhythmia [49].

In a recent review of 670 patients receiving sunitinib or sorafenib for renal cell carcinoma (all patients over 65 years old), there were 171 total cardiovascular events reported. The vast majority (146) were considered congestive heart failure or cardiomyopathy. This study demonstrates that there is likely a significant cardiac risk associated with use of TKIs, specifically sunitinib and sorafenib, however the study does not describe characteristic echocardiogram findings of TC in these patients [50]. In one study using a zebrafish model, it was suggested that both sorafenib and sunitinib lead to cardiomyocyte apoptosis, a reduction in total myocyte number per heart, contractile dysfunction and ventricular dilatation [51]. The authors hypothesized that the inhibition of the Raf/MEK/ERK pathway by sorafenib directly mediates the cardiotoxic effects of the drug, and that the alpha-adrenergic signaling pathway may play an important protective role against sorafenib cardiotoxicity [51].

Although cardiotoxicity associated with TKI use has been increasingly reported, it appears that TC associated with TKI use is a less commonly reported phenomenon. As demonstrated above for pazopanib, TC has been associated rarely with TKIs [45]. In the literature, TC has also been reported to be associated with sunitinib, with the characteristic apical ballooning pattern described on echocardiography [52]. A case of TC in association with axitinib has also been described [53]. One study postulated that VEGF antagonism and the following downstream effect via inhibition of endothelial nitric oxide synthase and nitric oxide, may inhibit physiological vascular responses to injury. The effects of nitric oxide on myocardial contractility and vascular tone may also contribute. In addition, nitric oxide can act as a modulator of catecholamine effects, at times accentuating it, and possibly contributing to the development of TC [45].

In the package insert for lenvatinib, cardiac dysfunction (decreased left or right ventricular function, cardiac failure, or pulmonary edema), was reported in 7% (17 cases) of lenvatinib-treated patients compared with 2% in the placebo group [41]. There is no specific comment on the possibility of TC. Therefore, to our knowledge the patient presented here is the first reported case of lenvatinib-associated TC. Interestingly, this patient developed two of the less-commonly reported adverse effects associated with lenvatinib use, and it is possible that the combination of both adverse effects is associated with an increased mortality.

## CONCLUSIONS

As targeted therapy with TKIs continues to increase in frequency, it is important to understand the potential adverse effects associated with such treatments. Two rare adverse effects, PRES and TC, were demonstrated in this patient treated with lenvatinib for radioiodine-refractory thyroid cancer and breast cancer. Although prior reports of these adverse effects with the use of other TKIs provides insight into their management, their rare nature limits providers’ ability to understand how to optimally manage them. This case report therefore provides additional insight to inform practice for prescribing oncologists, specifically with increasing awareness of these potential adverse effects so that truly informed decisions can be made when choosing TKI therapies. Providers should be aware of the variable time course of presentation of these adverse effects. Further reports are needed in order to elucidate risk factors for the development of these adverse effects, and further studies into potential mechanisms will provide a better understanding of the risks and benefits associated with these medications.

## Abbreviations

EBRT: external beam radiation therapy
EF: ejection fraction
LV: left ventricular
PRES: posterior reversible encephalopathy syndrome
RPLS: reversible posterior leukoencephalopathy syndrome
TC: takotsubo cardiomyopathy
TKI: tyrosine kinase inhibitor
TTE: transthoracic echocardiogram
VEGF: vascular endothelial growth factor.
